# Disparities in hypertension among black Caribbean populations: a scoping review by the U.S. Caribbean Alliance for Health Disparities Research Group (USCAHDR)

**DOI:** 10.1186/s12939-015-0229-0

**Published:** 2015-11-05

**Authors:** Aurelian Bidulescu, Damian K. Francis, Trevor S. Ferguson, Nadia R. Bennett, Anselm J. M. Hennis, Rainford Wilks, Eon N. Harris, Marlene MacLeish, Louis W. Sullivan, on behalf of the U.S. Caribbean Alliance for Health Disparities Research Group (USCAHDR)

**Affiliations:** Department of Epidemiology and Biostatistics, Indiana University School of Public Health - Bloomington, Bloomington, Indiana USA; Epidemiology Research Unit, Tropical Medicine Research Institute, The University of the West Indies, Kingston, West Indies Jamaica; Chronic Disease Research Centre, Tropical Medicine Research Institute, The University of the West Indies, Bridgetown, West Indies Barbados; The University of the West Indies, Kingston, West Indies Jamaica; Department of Medical Education, Morehouse School of Medicine, Atlanta, Georgia; The Sullivan Alliance, Alexandria, Virginia USA

## Abstract

**Background:**

Despite the large body of research on racial/ethnic disparities in health, there are limited data on health disparities in Caribbean-origin populations. This scoping review aimed to analyze and synthesize published and unpublished literature on the disparities in hypertension and its complications among Afro-Caribbean populations.

**Methods:**

A comprehensive protocol, including a thorough search strategy, was developed and used to identify potentially relevant studies. Identified studies were then screened for eligibility using pre-specified inclusion/exclusion criteria. An extraction form was developed to chart data and collate study characteristics including methods and main findings. Charted information was tagged by disparity indicators and thematic analysis performed. Disparity indicators evaluated include ethnicity, sex, socioeconomic status, disability, sexual orientation and geographic location. Gaps in the literature were identified and extrapolated into a gap map.

**Results:**

A total of 455 hypertension related records, published between 1972 and 2012, were identified and screened. Twenty-one studies met inclusion criteria for detailed analysis. The majority of studies were conducted in the United Kingdom and utilized a cross-sectional study design. Overall, studies reported a higher prevalence of hypertension among Caribbean blacks compared to West African blacks and Caucasians. Hypertension and its related complications were highest in persons with low socioeconomic status. Gap analysis showed limited research data reporting hypertension incidence by sex and socioeconomic status. No literature was found on disability status or sexual orientation as it relates to hypertension. Prevalence and morbidity were the most frequently reported outcomes.

**Conclusion:**

The literature on hypertension health disparities in Caribbean origin populations is limited. Future research should address these knowledge gaps and develop approaches to reduce them.

**Electronic supplementary material:**

The online version of this article (doi:10.1186/s12939-015-0229-0) contains supplementary material, which is available to authorized users.

## Background

Hypertension is an important worldwide public health challenge because of its high prevalence and concomitant risks of cardiovascular and kidney disease [1]. It has been identified as the leading risk factor for mortality and is ranked third as a cause of disability-adjusted life-years [2]. As presented in the study by Kearney and colleagues, the estimated total number of adults with hypertension in 2000 was 972 million (with an estimated confidence interval of 957 to 987 million), a third of which was in developed countries, and 639 million (625–654 million for the estimated confidence range) in economically developing countries [3]. A review by Barcelo indicated that the number of adults with hypertension in 2025 was predicted to increase by about 60 % to a total of 1.56 billion (1.54 to 1.58 billion, as the confidence interval) [4]. In Latin America and the Caribbean approximately one half of the 60 years and older population are living with hypertension [5]. In Barbados, the prevalence of hypertension for those aged 40 years and over was 55 % in 2002; 37 % of cases were undiagnosed, and only 34 % of those taking hypertension treatment achieved target blood pressures (<140/90 mm Hg) [6]. The prevalence of hypertension was reported as being higher in the Latin America and the Caribbean, compared to other geographic regions such as Asia and non-black or Hispanic Asian Islands [3].

These differences are in keeping with the emerging body of literature highlighting growing disparities in health over the last twenty years. According to the Minority Health and Disparities Research and Education Act United States public law 106–525 (2000, p. 2498) a health disparity exist where *“there is a significant disparity in the overall rate of disease, incidence, prevalence, morbidity, mortality or survival rates in the population as compared to the health status of the general population”.* Research recognizes that patterns in allocation of resources and differential access to care are a part of the broader systems that may influence health in population sub-groups [7]. In 2011, the World Health Organization (WHO) convened a World Conference on Social Determinants of Health and issued a political declaration expressing global political commitment for the implementation of social determinants of health approach to reduce health inequities [8].

Fundamental questions that must be asked when addressing health disparities are: “why are some people healthy and others not?” or “why do two people with the same disease have a different outcome?” Public health research has shown that for there to be an improvement in the health of a population, the social and economic disadvantages that are found in groups within societies as well as the political infrastructure, health policies and legal framework of a country must be addressed [7, 9]. A large body of research has now shown that health follows a socioeconomic gradient in which populations or groups with lower socioeconomic status experience higher rates of disease and poorer health outcomes [10], and that health is consistently worse for individuals with few resources [11–13]. However the construct of health disparity is much more complex and other factors such as the social and cultural practices/norms also must be considered, and the contribution of each [14].

The Caribbean is a geographically diverse region with a predominantly black population with its citizens living both inside and outside of the region, including the United States. The racial admixture varies between countries; for example, in Jamaica and Barbados over 90 % of the population is of African descent while in Trinidad and Tobago and Guyana over 50 % of the population are of South Asian origin or mixed ethnicity. In the Caribbean, the overall age-adjusted prevalence of hypertension among persons 25 years and older ranges from 41 – 47 % based on data derived from the WHO Global Health Observatory (Ferguson, personal communication). For persons 15–74 years old in Jamaica, national prevalence of hypertension is estimated at 25 % [15]. Hypertension is thus one of the major public health challenges for the Caribbean in the twenty-first century.

Although it is assumed that the factors which account for the disparities seen within Caribbean populations are the same as elsewhere, no literature review exists to verify or refute this supposition. This study therefore aimed to conduct a review of published studies of hypertension in Caribbean populations in order to identify the extent of available literature and to determine which factors drives the disparities observed. The specific objectives were:

1. To synthesize the published evidence on health disparities in hypertension among Caribbean origin populations;
2. To evaluate the reported health disparities in the incidence and prevalence of hypertension, complications of hypertension, and mortality related to hypertension;
3. To identify which health disparity indicators are more frequently reported among Caribbean populations and identify gaps in the literature on health disparities in hypertension.

Scoping reviews have emerged recently as a method which “aims to rapidly map the key concepts underpinning a research area and the main sources and types of evidence available. It can be undertaken as stand-alone projects, especially where an area is complex or has not been reviewed comprehensively before” [16, 17]. Like the systematic review, but unlike other traditional literature reviews, the scoping review employs a systematic replicable approach which includes a search strategy to reduce bias. Nevertheless, we did not assess the methodological quality of the included studies in this scoping review.

## Methods

### Inclusion criteria

Studies that reported on health disparities (stratified by age, sex, ethnicity/race, geographic location, sexual orientation, disability status and socioeconomic status) in hypertension among Caribbean populations using all study designs were examined. This was part on a larger project that identified a series of chronic conditions including diabetes mellitus, cardiovascular diseases, hypertension, chronic obstructive pulmonary disease, asthma, cancer and depression, described in details in our previous publication [18]. From our search of published and unpublished literature all citations were entered into the reference manager Endnote and of these 455 articles were found to potentially be relevant to hypertension as well as a common core for all the chronic conditions searched.

Disability status was defined as a permanent physical or mental inability to carry out routine functions, and socioeconomic status was measured by occupation, education, income, or household amenities. Study participants had to be adults 18 years or older, of African descent, living in the any Caribbean country (including Belize, Guyana and Suriname; see Additional file 1: Appendix 1 for complete list of countries included) or part of the Caribbean immigrant populations of African descent living outside of the Caribbean.

### Exclusion criteria

We excluded studies which did not report on an Afro-Caribbean population or immigrant populations of Caribbean descent alone or as a comparator group with other populations (e.g. African American, UK-Africans). Studies which grouped the Afro-Caribbean population with another ethnic group, e.g. West African or Latin American, so that separate effects could not be obtained were excluded, as were studies in which the < 18 year old age group could not be separated from the ≥ 18 year old age group.

### Types of outcome measures

Both absolute and relative outcome measures (including incidence, prevalence, risk ratio, odds ratio) were extracted as well as any additional descriptive qualitative-type information found.

As health disparities can occur by gender race or ethnicity, education or income, disability, living in rural localities or sexual orientation, we chose these disparity indicators to be used for our study. Through measurement of the indicators of health, the degree of disparity can be also characterized by absolute and relative differences in measures of occurrence captured as proportions, rates and ratios. These are defined as disparity measures, measures that we only summarized across our review, but were not the main topic of our project.

### Search strategy

A comprehensive search strategy was developed in consultation with an information specialist. The search was designed to retrieve all articles combining the concepts of ‘Caribbean region’, ‘African ancestry’ and ‘black Caribbean ethnicity’ with specific chronic diseases (diabetes mellitus, hypertension, cardiovascular diseases, pulmonary diseases, cancer, and depression; from which we specifically selected the hypertension papers), and social determinants of health, health disparities, or health inequity in relevant bibliographic databases. The following databases were searched:

- Ovid MEDLINE(R) <1946 to June 20, 2013>)
- Ovid MEDLINE(R) In-Process & Other Non-Indexed Citations <1946 to June 20, 2013>)
- CENTRAL via Cochrane Library (February 2013)
- LILACS
- PsycINFO 1806 to June Week 3 2013.

For conference proceedings, theses, dissertations and other grey literature the following databases were searched:

- Science Citation Index Expanded (SCI-EXPANDED) --1992-present
- Social Sciences Citation Index (SSCI) --1992-present
- Arts & Humanities Citation Index (A&HCI) --1992-present
- Conference Proceedings Citation Index- Science (CPCI-S) --1992-present
- Conference Proceedings Citation Index- Social Science & Humanities (CPCI-SSH) --1992-present
- ProQuest: Theses and Dissertation 1990-present.

The search was conducted without a study design filter in order to retrieve qualitative as well as quantitative papers. The search was limited to publications in the English language as was appropriate to our population. The original search strategy was modified and updated twice over a two year period to accommodate adjustments in the concepts of population including countries defined as Caribbean based on the results of the initial search strategy and consultation with Caribbean research experts and information scientists.

### Review process

#### Screening and charting

Duplicate articles were identified and removed from the database prior to screening. The titles and abstracts of articles identified by the search strategy were independently screened for relevance by two review authors according to the inclusion and exclusion criteria described above. Citations were managed using Endnote X5 and Microsoft Excel. Discrepancies between review authors were resolved through discussion and, where necessary, by consultation with a third review authors. Studies meeting the criteria outlined were charted using a standard study extraction form with domains as listed in Additional file 1: Appendix 2. Six (6) of the studies were actual theses which were not accessible through our library and our search revealed no published studies resulting from these thesis specific to hypertension. The other two (2) studies were journal articles published in 1972 and 1979 which were not yielded when ordered from the BIREME library systems for Latin America and the Caribbean via our local library. We are not of the view that these publications would significantly reshape the results and or inferences of this review.

### Data synthesis

Data synthesis was conducted following approach outlined Arsky and Omalley [19]. Quantitative data was analyzed to demonstrate the characteristics and distribution of the included studies in the review. The data was represented in tabular forms as well as charts outlining the distribution of studies according to geographic location, study design, publication year, outcome measures used to address disease entity, and disparity indicators. Resulting from this process knowledge gaps were identified and graphically displayed in a gap chart. Thematic synthesis included describing charted data organized according to the a priori themes (disparity indicators) as well as emerging areas such as health care utilization and hypertension risk factors. The results of this review are therefore categorized according to 1) prevalence of hypertension, 2) incidence of hypertension, 3) mortality from hypertension, 4) morbidity and mortality of hypertension complications, and 5) healthcare utilization and access.

## Results

### Characteristics of included studies

**Fig. 1:**
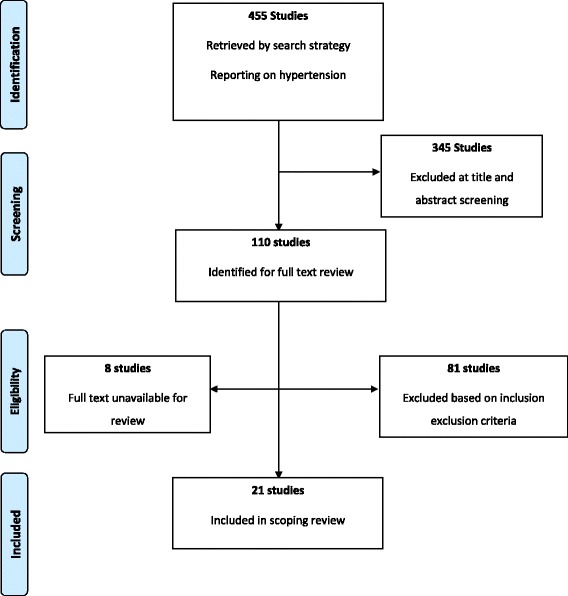
Summary of the inclusion and exclusion process

**Fig. 2:**
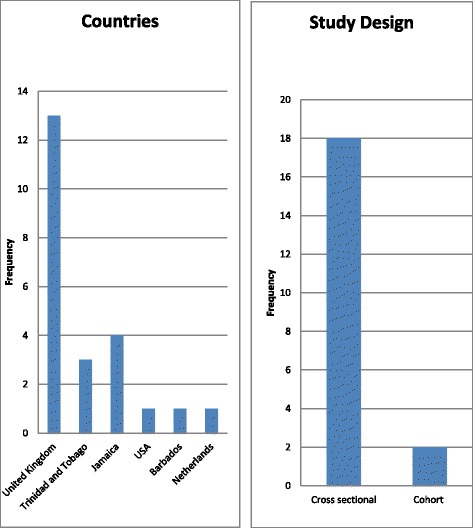
Distribution of included studies by study location and design

455 studies which reported on hypertension (also described in the literature as high blood pressure) and its related health disparities were identified from the overall search. After title and abstract screening of these references 110 studies required full text review from which 21 studies were selected for final analysis (Fig. 1). The majority of studies were conducted in the United Kingdom and of cross sectional study design (Fig. 2). Studies were mainly population based and included comparisons of Afro-Caribbeans to South-Asians, Caucasians, and African blacks (Table 1).

**Table 1:** Characteristics of studies included in analysis

Author	Year	Ethnic group (Location)	Country	Study Design	Setting	Disparity measure	Theme
Agyemang	2010	South East Asian Indians vs. Afro-Caribbean in England and Netherlands	United Kingdom, Netherlands	Cross-sectional study	Population-based	Ethnicity	Prevalence of HTN
Agyemang	2012	Caucasians and Afro-Caribbean	United Kingdom	Cross-sectional	Population-based	Age	Prevalence of HTN
Baskar	2006	Afro-Caribbean vs. Caucasians vs. Indo-Asians	United Kingdom	Cross-sectional	Population-based	Ethnicity	Prevalence of HTN and complications
Birns	2008	Caucasians vs. Black African origin	United Kingdom	Cross-sectional study	Hospital based	Ethnicity	Complications from HTN
Chaturvedi	1993	European Whites vs. Afro-Caribbeans	United Kingdom	Cross-sectional	Clinic-based	Ethnicity	Prevalence of HTN
Chaturvedi	2004	Afro-Caribbean vs. European (UK)	United Kingdom	Cross-sectional	Community-clinic based	Ethnicity	Prevalence of HTN complications
Conway	2003	Afro-Caribbean vs. European vs. South Asian	United Kingdom	Cross-sectional	Hospital-based	Ethnicity	Prevalence of HTN and Atherosclerotic Vascular disease
Cooper	1997	West Africa (Nigeria and Cameroon) vs. Caribbean (Jamaica, St. Lucia, Barbados) vs. the U.S. (metropolitan Chicago, Illinois)	U.S., Caribbean	Cross-sectional	Population-based	Ethnicity	Prevalence of HTN and its related risk factors
Cruickshank	2001	African (urban vs. rural) vs. Caucasian (urban vs. rural)	United Kingdom, Jamaica	Cross-sectional study	Population-based	Ethnicity, Geographic location	Prevalence of HTN
Fang	1996	NE whites vs. South blacks vs. NE blacks vs. Caribbean blacks	United States	Cross-sectional	Community-based	Ethnicity, Geographic location, and Sex	Mortality from HTN
Ferguson	2011	Caribbean blacks	Jamaica	Cross-sectional	Community-based	Sex, and Age	Prevalence of HTN
Gulliford	2004	Afro-Trinidadian vs. Indo-Trinidadian vs. mixed vs. other	Trinidad and Tobago	Cross-sectional	Community-based	Socioeconomic Status and Sex	Prevalence of HTN
Lane	2002	African-Caribbean vs. Caucasians vs. South-Asians	United Kingdom	Cross-sectional	Population-based	Age, Ethnicity, and Sex	Prevalence of HTN
Markus	2007	Whites vs. Black individuals	United Kingdom	Cross-sectional	Clinic-based	Ethnicity	Prevalence of HTN
Mendez	2003	Blacks	Jamaica	Cross-sectional	Population-based	Socioeconomic status, and Sex	Prevalence of HTN
Miller	1996	African ancestry vs. African-European ancestry vs. Indian ancestry	Trinidad and Tobago	Cohort study	Population-based	Ethnicity and Sex; BMI	Incidence of HTN
Molokhia	2000	Afro-Caribbean	United Kingdom	Cross-sectional	Clinic-based	Ethnicity	Prevalence of HTN
Molokhia	2011	Afro-Caribbean	Trinidad and Tobago	Cohort study	Population-based	Sex	Prevalence of HTN
Morris	2011	Black vs mixed	Jamaica	Cross sectional	Population based	Geographic location	Prevalence of HTN
Sedgwick	2003	African blacks and Caribbean blacks	United Kingdom	Cross sectional	Clinic-based	Ethnicity	Prevalence of HTN among persons with Diabetes
Stewart	2002	African-Caribbean	United Kingdom	Cross sectional	Clinic-based (secondary data analysis)	Socioeconomic status (education/school leaving age)	Prevalence of HTN

### Disparities in the prevalence of hypertension

Overall the prevalence of hypertension was found to be higher in Afro-Caribbeans compared to Caucasians, South-Asians and African blacks in the Cameroon [15, 20–30]. One study reported that at around 30 and 40 years of age systolic blood pressure values in men and diastolic blood pressure values in men and women increase in African-Caribbean people steeper than in their white counterparts [20]. However, there were mixed results when Afro-Caribbeans were compared to African counterparts. Three studies found the prevalence of hypertension to be higher in Afro-Caribbeans compared to African blacks [20, 25, 31]. On the other hand in a multi-ethnic clinic based sample living in the UK, Chaturvedi reported that the prevalence of hypertension was similar among Afro-Caribbeans and their West African counterparts [23]. Cooper and colleagues [32] found that a consistent gradient of hypertension prevalence was observed, rising from 16 % in West Africa to 26 % in the Caribbean and 33 % in the United States. Mean blood pressures were similar among persons aged 25 to 34, while the increase in hypertension prevalence with age was twice as steep in the U.S. as in Africa [32].

A total of eight studies reported on age [15, 20, 27] and sex [15, 26–28, 33] disparities in the prevalence of hypertension. Sex differences were mainly explored through sub-group analysis within studies reporting on other disparities in hypertension among multiethnic populations. Ferguson and colleagues in a nationally representative survey in Jamaica reported that there were no differences in prevalence of hypertension by sex [15], while two studies reported a higher burden of hypertension among Afro-Caribbean women compared to men [27, 33]. In contrast, two studies found that the prevalence of hypertension was higher in men compared to women with the difference ranging from 4 % [34] to 11 % [35]. As a sub-theme the prehypertension, examined by one study, was notably higher in men when compared with women residing in Jamaica [15].

All three studies reporting on age [15, 20, 27] found that hypertension increased with age among the Afro-Caribbean ethnic group similar to other ethnic groups. However, one study noted that Afro-Caribbean men less than 40 years of age had a lower prevalence of hypertension compared to South Asians men in a similar age category [27]. In addition, systolic blood pressure in men, and diastolic blood pressure in men and women appeared higher in African-Caribbean people compared to white counterparts in the 30 and 40 years age category [20].

Social disparities in the prevalence of hypertension were examined by three studies [26, 36, 37]. In Trinidad and Tobago, a study by Gulliford found a noticeable decrease in the prevalence of hypertension as monthly household income increased in both sexes but it was only statistically significant in women (*p* = 0.04). Hypertension was also inversely associated with social capital in terms of income and education but this relationship was only observed in women. Additionally, all three studies demonstrated that the burden of hypertension was higher among the lesser educated; in Jamaica the gap in prevalence was as wide as 64 % between the educated and lesser educated group (college/university, 5.4 %; less than 6^th^ grade or vocational, 69.6 %) [36].

**Fig. 3:**
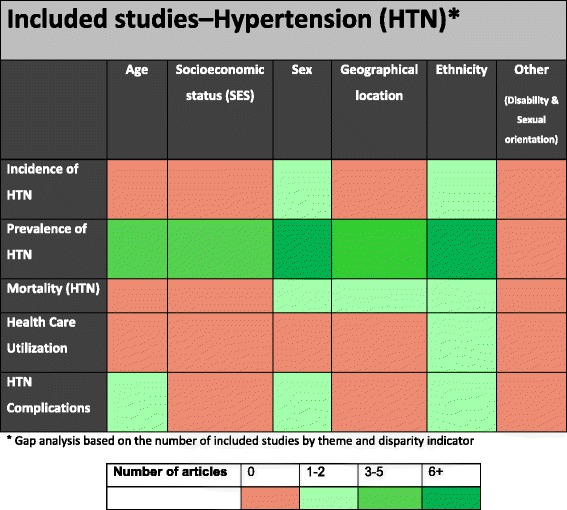
Health disparities research gaps identified in hypertension literature

When the prevalence of hypertension by geographic location was considered, Cruickshank and colleagues [25], found age-adjusted rates of hypertension ranging from 5 % in rural to 17 % in urban Cameroon, despite young mean ages, whereas it was 21 % in Jamaica and 34 % in Caribbean migrants to Britain, the majority of whom were Jamaicans. In a study conducted in Jamaica, self-reported hypertension was higher among rural participants compared to those living in urban areas (60.1 % vs. 39.9 %) [29]. There were no studies that looked at the prevalence of hypertension in the Afro-Caribbean ethnic group by disability status and or sexual orientation (Fig. 3).

### Disparities in the incidence of hypertension

Ethnicity had no significant influence on the incidence of hypertension across the Afro-Caribbean population of Trinidad and Tobago ranging between 33 and 41 per 1000 person-years in men and between 27 and 32 per 1000 person-years in women [38]. One identified sub-theme suggests that the incidence of hypertension increased with body mass index [38].

### Hypertension related mortality

One study investigated mortality attributable to hypertension relating to some measure of health disparity. There were substantial differences in death rates among blacks according to birthplace. In the study by Fang and colleague [39], standardized mortality rates (SMR) were higher in Caribbean born blacks compared to whites in the United States. However, the SMR was lower among the Caribbean born ethnic group compared to US-born blacks in both Northeast and South US [39]. Caribbean born men and women had a lower mortality rate from hypertension compared to US-born blacks except for in the 65 years and older group (Northeast US born blacks only). Hypertension related mortality also increased with age [39].

### Disparities in hypertension related complications

Four studies examined differences in hypertension related complications including cognitive dysfunction, microvascular and macrovascular complications [22, 24, 40, 41]. There are ethnic differences in vascular remodeling that would provide valuable clues to understanding the consequences of hypertension; it seems that the elastic aorta is stiffer in African-Caribbean compared to Europeans [41]. In terms of vessels affected, in blacks, 33 % of strokes were due to cerebral small vessel disease compared with 14 % in whites. Blacks had less large vessel atherosclerosis and cardio-embolic disease [31]. Atherosclerotic vascular disease was found to be higher in whites compared to Afro-Caribbeans but this difference was not statistically significant [24]. Relative to the Caucasian group, the Afro-Caribbean group had higher prevalence of hypertension and related microvascular complications but lower macrovascular disease [22]. In addition, microvascular and macrovascular complications among Afro-Caribbeans were similar to South-Asians. In another study conducted in the United Kingdom, Afro-Caribbean showed increased white matter damage and executive cognitive dysfunction compared with Caucasians after adjustment for age, blood pressure level and treatment, duration of hypertension and vascular risk factor profile [40].

### Healthcare utilization and access among persons with hypertension

In a single study that directly examined healthcare utilization and access among persons with hypertension, overall healthcare utilization was higher among Afro-Caribbeans compared to Caucasians particularly for treatment of elevated blood pressure, self-monitoring (blood /urine), and number of visits to Diabetes Nurse and Dietitian [38].

### Disability and sexual orientation

Although disparities in hypertension as it relates to disability and sexual orientation were of interest, no studies in this area were found. We found no articles relating hypertension to the two health disparity indicators, despite the fact that (physiologically) one would expect disability to increase hypertension prevalence (due mainly to physical inactivity). In addition both sexual orientation and disability may limit access to care through social barriers (discrimination, behavioral characteristics, etc.) which were explored in this review. Disability and sexual orientation may also limit access to care which was explored in our review, as well as affect adverse outcomes.

## Discussion

### Summary of findings

Overall from the 21 studies included in this systematic review the prevalence of hypertension is higher among those with Afro-Caribbean ethnicity when compared to Caucasians, South-Asians and African blacks. The literature was less clear on the sex differences in hypertension with the similar number of studies reporting a higher prevalence in men compared to women and *vice versa*. The prevalence of hypertension increased with age, the blood pressure in both men and women is higher at 30–40 years of age in African-Caribbeans compared to their Caucasian counterparts. The low all-cause mortality for Caribbean immigrants and the excess mortality from hypertensive diseases in migrants from both West Africa and the Caribbean suggest that socio-genetic factors may underlie the susceptibility to hypertension in people of black African descent [39, 40]. The burden of hypertension follows a social gradient with poorer and less educated individuals having a higher prevalence of the disease.

### Health disparities versus degree of disparity

Health disparities term is defined in the literature as the variation or differences in health status resulting from the distribution of the effects of health determinants between and among different population groups [41]. In addition health disparities imply a social disadvantage among population-subgroups as it relates to a particular health outcome such, as morbidity, mortality or access to care. These health disparities can occur by gender race or ethnicity, education or income, disability, living in rural localities or sexual orientation [42]; disparity indicators that we have used for our current study. Through measurement of the indicators of health, the degree of disparity can be also characterized by absolute and relative differences in measures of occurrence captured as proportions, rates and ratios. These are defined as disparity measures, measures that we only summarized across our review, as they were provided by the authors.

### Correlates for health disparities in hypertension among Caribbean populations

Our scoping review points mainly to ethnicity, sex, and geographical location as main correlates of hypertension prevalence, with age in close vicinity. Age, sex, and ethnicity appear also as main correlates for hypertension complications, whereas sex, ethnicity and geographical location are correlates for hypertension mortality. From a mechanistic perspective, the main factors appear to be chronic stress, discrimination, lack of control as well as behavioral factors such as nutritional intake and physical activity levels, well aligned with what is found in the literature [43]. Thus, mechanism related to ethnicity, geographical disparities, social factors and aging status appear as main factors that should be addressed in order to better understand and reduce these disparities. The main risk factors for hypertension, such as obesity and salt intake [32], were not adequately explored across the comparison groups in the reviewed articles, but probably will explain the bulk of the disparities alongside socioeconomic status. The latter remains a “black box” which needs to be explored in all future work on disparities.

### Overall completeness and applicability of review

This review presents an in-depth outline of the scope of the literature published from 1970s onwards, relating to the investigation of disparities in the hypertension in the Caribbean and the Caribbean Diaspora. The review was based on a comprehensive search of the literature and as such should capture the full range of available studies on health disparities in the Caribbean. Most studies were done in an urban setting in a community or hospital health clinic on Afro-Caribbean immigrants to the United Kingdom over a time period from 1972–2012. The comparison groups were between Afro-Caribbean groups with Caucasians, African Caribbean and Asians with many different ethnicities; very few studies were compared to the Latin American population.

The findings of higher burden of hypertension among Afro-Caribbean compared to other ethnic groups in this review posits whether prevention and screening strategies would be effective in reducing this disparity if instituted earlier in African-Caribbean populations. This principle is supported by Kurian and colleagues in a systematic review examining racial and ethnic disparities in cardiovascular disease risk factors [44]. The finding of a consistently higher prevalence of hypertension among Afro-Caribbeans compared to other ethnic groups are reconcilable with those of Kearney et al. [45], where in countries with predominantly multi-ethnic populations such as the United States, hypertension was more prevalent in blacks than whites. The lack of a clear finding on sexual differences in hypertension among Afro-Caribbeans is less consistent with what was hypothesized as only three studies found a higher burden among men than women. In the worldwide prevalence of hypertension systematic review by Kearney, hypertension was most prevalent in men compared to women across ethnicity [45].

There are however several gaps in the current knowledge on disparities in hypertension. The few studies that stated that hypertension is inversely associated with social capital mirrors differences seen between American Indians and non-Hispanic Whites in a recent systematic review [46]. In addition, although disparities in hypertension as it relates to disability and sexual orientation may be of interest, especially as it relates to access to care and outcomes, no studies in this area were found.

### Potential biases in the review process

Though the search strategy of this review was iterative and broad, potentially relevant studies may have been excluded due to a language filter to only include studies published in English language. It is however, well documented that though studies on Spanish and French speaking Caribbean as well as Caribbean immigrant populations are published in other languages, the predominant language for publication is English [47, 48]. In addition there were potentially eight studies that were retrieved by our search strategy that could have contributed information had the full text reviews been available for analysis. A potential limitation is that this scoping review did not include an assessment of methodological quality of the included studies. This is however in keeping with approaches for conducting scoping reviews as they are largely aimed at 1) summarizing literature, 2) identifying gaps more studies including systematic reviews are warranted and 3) recommendations for policy depending on the nature of the review [49].

## Conclusions

We have found that while a number of studies have been published exploring health disparities in relation to hypertension, the literature on hypertension health disparities in Caribbean-origin populations is limited in particular as it relates to studies conducted within the Caribbean. There were very few studies comparing Afro-Caribbean populations with African Americans, an ethnicity of interest within the United States. Such studies would help in understanding the mechanisms underlying health disparities among minority population in the United States and the influence of factors self-governance, discriminations and variations in health care systems on health disparities. Future research should address these knowledge gaps and approaches to reduce them as we seek to reduce health disparities and improve health for all social and ethnic groups.

## Additional file

Additional file 1: Search and Data Extraction Strategy (DOCX 26 kb)
