# Supplementary material for: Disparities in hypertension among black Caribbean populations: a scoping review by the U.S. Caribbean Alliance for Health Disparities Research Group (USCAHDR)
Source: Int J Equity Health. 2015 Nov 5;14:125. doi: 10.1186/s12939-015-0229-0 (PMC4635613; doi:10.1186/s12939-015-0229-0)
Supplement: Additional file 1: — Search and Data Extraction Strategy (DOCX 26 kb) [file 12939_2015_229_MOESM1_ESM.docx]

***Appendix 1:***

***Search Strategy***

Database: Ovid MEDLINE(R) In-Process & Other Non-Indexed Citations and Ovid MEDLINE(R) <1946 to Present>

Search Strategy:

--------------------------------------------------------------------------------

1 Caribbean/eh (575)

2 African Americans/eh, ge (3174)

3 Caribbean Region/eh (575)

4 African Continental Ancestry Group/eh, mo, ed, sn (2998)

5 Lucayan Archipelago/ (0)

6 Bahamas/ (309)

7 Turks.mp. and Caicos Islands/ [mp=title, abstract, original title, name of substance word, subject heading word, keyword heading word, protocol supplementary concept, rare disease supplementary concept, unique identifier] (0)

8 Turks Island/ (0)

9 Caicos Island/ (0)

10 Greater Antilles/ (0)

11 Cuba/ (4051)

12 Hispaniola/ (0)

13 Haiti/ (2172)

14 Dominican Republic/ (1079)

15 Jamaica/ (2848)

16 Cayman Islands/ (3153)

17 Puerto Rico/ (4798)

18 Lesser Antilles/ (0)

19 Leeward Islands/ (0)

20 United States Virgin Islands/ (180)

21 US Virgin Islands/ (0)

22 Saint Croix/ (0)

23 Saint Thomas.mp. (39)

24 Saint John/ (0)

25 Water Island/ (0)

26 British Virgin Islands/ (3153)

27 Tortola/ (0)

28 Virgin Gorda/ (0)

29 Anegada/ (0)

30 Jost Van Dyke/ (0)

31 Anguilla/ (1289)

32 Antigua/ (82)

33 Barbuda/ (82)

34 Redonda/ (0)

35 Saint Martin/ (0)

36 Sint Maarten/ (0)

37 Saba/ (0)

38 Sint Eustatius/ (0)

39 Saint Barthelemy/ (0)

40 Saint Barth*lemy/ (0)

41 Saint Kitts/ (44)

42 Nevis/ (44)

43 Montserrat/ (3153)

44 Guadeloupe/ (292)

45 French Antilles/ (0)

46 Les Saintes/ (0)

47 Marie*Galante/ (0)

48 La Desirade/ (0)

49 La D*sirade/ (0)

50 Windward Islands/ (0)

51 Dominica/ (64)

52 Martinique/ (366)

53 Saint Lucia/ (45)

54 St Lucia/ (45)

55 "Saint Kitts and Nevis"/ (44)

56 Saint Vincent/ (35)

57 St Vincent/ (35)

58 Grenadines/ (35)

59 Grenada/ (77)

60 Carriacou/ (0)

61 Petite Martinique/ (0)

62 Barbados/ (499)

63 Trinidad/ (1370)

64 Tobago/ (1370)

65 Leeward Antilles/ (0)

66 Aruba/ (3153)

67 Curacao/ (0)

68 Bonaire/ (0)

69 West Indies/ (3153)

70 West Indian/ (0)

71 West India/ (0)

72 West Indi*/ (0)

73 St thomas.mp. (1261)

74 St John.mp. (296)

75 St Martin.mp. (49)

76 st Maarten.mp. (13)

77 Netherlands Antilles/ or st Eustatius.mp. (256)

78 st Barthelemy.mp. (12)

79 st Kitts.mp. or "Saint Kitts and Nevis"/ (124)

80 "Virgin Islands of the United States"/ or St Croix.mp. (278)

81 Guyana/ or Guyana$.tw. (837)

82 Suriname/ or Surinam$.tw. (1182)

83 (French adj Guyana).mp. (184)

84 Belize/ or Belize.mp. (618)

85 anguilla$.tw. (2680)

86 (antigua adj2 barbuda).tw. (32)

87 antigua$.tw. (160)

88 barbud$.tw. (41)

89 aruba$.tw. (90)

90 bonaire.tw. (28)

91 curacao$.tw. (247)

92 baham$.tw. (550)

93 barbad$.tw. (1089)

94 beliz$.tw. (512)

95 bermud$.tw. (1052)

96 tortola$.tw. (28)

97 curacao.tw. (244)

98 (virgin adj2 gorda).tw. (0)

99 anegad$.tw. (4)

100 cayman$.tw. (166)

101 cuba$.tw. (5421)

102 dominica$.tw. (1863)

103 grenad$.tw. (430)

104 grenad$.tw. (430)

105 haiti$.tw. (2521)

106 jamaica$.tw. (3308)

107 montserrat$.tw. (86)

108 (puerto adj2 rico).tw. (4160)

109 martiniqu$.tw. (526)

110 guadelop$.tw. (4)

111 saint kitts-nevis.tw. (1)

112 (nevis$ or kitt$).tw. (3904)

113 lucia$.tw. (573)

114 eustatius.tw. (8)

115 (maarten$ or martin$).tw. (6867)

116 barthelem$.tw. (56)

117 saba.tw. (308)

118 trinidad$.tw. (1793)

119 tobago$.tw. (596)

120 (virgin adj2 island$).tw. (357)

121 USVI.tw. (34)

122 (thomas$ or croix$ or john$).tw. (98780)

123 (west adj2 indi$).tw. (3534)

124 caribbean$.tw. (8317)

125 leeward.tw. (117)

126 antill$.tw. (781)

127 windward.tw. (103)

128 guyan$.tw. (1019)

129 surinam$.tw. (1048)

130 guian$.tw. (1304)

131 hispaniol$.tw. (151)

132 doming$.tw. (599)

133 or/1-132 (160218)

134 *Cardiovascular Diseases/ (63802)

135 *Heart Diseases/ (38016)

136 *Diabetes Mellitus/ (60718)

137 diabetes.mp. (395222)

138 Hypertension/ (188116)

139 (high adj blood adj pressure).mp. (10187)

140 Stroke/ (54028)

141 Socioeconomic Factors/ (109574)

142 Health Status Disparities/ (6870)

143 Social adj2 determinants adj2 health).mp. (1045)

144 (gender or sex).mp. [mp=title, abstract, original title, name of substance word, subject heading word, keyword heading word, protocol supplementary concept, rare disease supplementary concept, unique identifier] (695238)

145 Ethnic Groups/ or ethnicity.mp. (69773)

146 Age Factors/ (364634)

147 Educational Status/ (38804)

148 Income/ (20840)

149 Occupations/ (16755)

150 socioeconomic.mp. (141152)

151 sexual orientation.mp. (2566)

152 (gay or homosexual or lesbian).mp. [mp=title, abstract, original title, name of substance word, subject heading word, keyword heading word, protocol supplementary concept, rare disease supplementary concept, unique identifier] (14244)

153 Poverty Areas/ or Poverty/ (29695)

154 Urban Population/ or Urban Health/ (60795)

155 Rural Health/ or Rural Population/ or Rural Health Services/ (66001)

156 or/157-171 (1223690)

157 133 and 156 and 172 (6881)

158 limit 173 to yr="2013" (166)

159 or/85-132 (147358)

160 175 and 156 and 172 (5081)

161 limit 176 to yr="1860 - 2012" (4942)

**Appendix 2:**

**Data Extraction Domains**

| **Field** | **Field Description and Instructions** | **Allowable Values** |
| --- | --- | --- |
| Study ID | Insert any of the following here: PMID (accession number) , doi, ISSN, ISBN | alphanumeric |
| First Author | Enter surname in lower case at all times using the following format. [surname][,][initial 1][initial2] | text |
| Publication year | Enter the year that the study was published from the drop down menu | text |
| Title | Enter the exact title of the paper as it appears in the journal | text |
| Study location | Enter the country or countries, in which the study was carried out | text |
| Study aims/objectives | Enter the objectives of the paper and after the entry, it should be stated if the objectives entered were [stated][inferred]or [not known] | text |
| **METHODOLOGY** | | |
| Study Design | Classify the study broadly as randomized or non-randomized studies. Non-randomized studies should be classified as 1. Controlled before-and-after-study 2. Interrupted-time-series study, 3. Historically controlled study, 4. Cohort study, 5. Case-control study 6. Cross-sectional study, 7. Case series (uncontrolled longitudinal study). 8. Other (indeterminate). For non-randomized studies that do not report specific study design two review authors will assess the study design. | text |
| Description | Enter population based, hospital-based, community-based from drop-down menu | text |
| study began | Enter information provided by the paper in the following (when all fields are available) [date]-[month]-[year] | text |
| study ended | Enter information provided by the paper in the following (when all fields are available) [date]-[month]-[year] | text |
| Selection | Enter the inclusion and exclusion criteria given by the study | text |
| Predictors | Enter the disease the paper addressed. Eg. Diabetes, lung cancer etc. | text |
| outcome | Enter the outcome that the paper addressed e.g. mortality or health care utilization | text |
| outcome measures | Enter the outcome measure that was used in the study e.g. odds ration or relative risk | text |
| disparity measures | Enter the disparity variable that was used in the paper e.g. socioeconomic status which should not be abbreviated, and if more specific variables e.g. education were used they should all be specified | text |
| **Population characteristics** | This section describes the baseline characteristics of the study population (frequency distribution/proportions). Where there are no information for subsections within this category the word [not stated] should be used. | |
| age range | Enter the age range of study participants | text |
| sex/gender distribution | Enter the total number of participants as well as the number and percentage of participants that are male in the following format *n(%)* | text |
| ethnicity/race | Enter the total number and percentage of participants by ethnicities that were defined in the studies (including non- afro Caribbean) in the following format *n*(%) | text |
| sexual orientation | Enter the total number and percentage of participants by sexual orientation that were defined in the studies (homosexual, hetero-sexual, trans-gendered, other/not specified) in the following format *n*(%) | text |
| disability status | enter the total number and percentage of the participants who were disabled n(%) (state definition of disability if present in manuscripts) | text |
| socioeconomic status | Enter which socioeconomic variable(s) was reported by the study as well as the number and percentage that fell into each sub-category (e.g. Education, Occupation, Income, etc.) | text |
| **MAIN FINDINGS** | If no information was specified in the next 5 sub-sections the word [not stated] should be used. Report both univariate (UV) and multivariate findings (MV). If multivariate state the other variables in the model | |
| age | Enter the results or findings relating to outcome or disparity measured reported/stratified by age group/range. | text |
| sex | Enter the results or findings relating to outcome or disparity measured reported/stratified by sex/gender (male vs female). | text |
| ethnicity/race | Enter the results or findings relating to outcome or disparity measured reported/stratified by ethnicity/race | text |
| location | Enter the results or findings relating to outcome or disparity measured reported/stratified by different area of residence (urban vs rural) | text |
| sexual orientation | Enter the results or findings relating to outcome or disparity measured reported/stratified by sexual orientation (homosexual, hetero-sexual, trans-gendered, and other/not specified). | text |
| disability status | Enter the results or findings relating to outcome or disparity measured/ stratified by disability as it was stated by the paper | text |
| socioeconomic status | Enter the results or findings relating to outcome or disparity measured reported/stratified by socioeconomic status composite or single components. | text |
| CONCLUSION | Enter the conclusion that was stated by the authors in the paper, if no conclusion was stated indicate this by [not stated] | text |
| LIMITATIONS STATED | Enter the limitations that were stated by the authors of the paper | text |
| OVERALL CONCLUSION ON STUDY | Enter reviewer overall conclusion on study based on authors conclusion and limitations of the study (stated or unstated) | text |
| SCOPING REVIEW CLASSIFICATION 1 | Click the drop down menu and select the option that best categorizes the paper | text |
| SCOPING REVIEW CLASSIFICATION 2 | Click the drop down menu and if a second category defines the paper select it | text |
| INCLUDE/EXCLUDE | Enter the word [include] or [exclude], to indicate whether or not the paper should be included in the scoping review | text |
| REVIEWED BY | Enter the initials of the review author that extracted the data | text |
